# 

**Published:** 1884-03

**Authors:** 


### BUREAUS OF A. H. A., 1884.

### OBSTETRICS AND DISEASES OF WOMEN AND CHILDREN.

Dr. J. R. Haynes, *Chairman*, Indianapolis; Drs. J. P. Mills, Chicago; A. McNeil, Jeffersonville, Ind.; J. A. Compton, Indianapolis; C. P. Beaman, Stanford, Conn.; Theo. Foote, Vineland, N. J.; Tuller, Vineland, N. J.

### BUREAU OF SURGERY.—SUBJECT FOR DISCUSSION, CONSTITUTIONAL EFFECTS OF INJURY.

C. H. Lawton, M. D., *Chairman*.

1. *Shock, Etiology and Diagnosis*, C. H. Lawton, M. D.
2. *Shock, Prognosis and Therapeutics*, Edward Cranch, M. D.
3. *Traumatic Fever and Delirium*, Edward Mahony, M. D.
4. *Traumatic Encephalitis*, L. B. Wells, M. D.
5. *Traumatic Erysipelas*, C. W. Butler, M. D.
6. *Tetanus, following an injury of the Spinal Nervous System*, H. I. Ostrom, M. D.
7. *Therapeutics* (*traumatic*), J. B. Bell, M. D.

### BUREAU OF MAT. MED., WITH PROVING OF LAC FELINUM.

E. Rushmore, *Chairman;* Drs. C. Lippe, E. Cranch, Laura Morgan, L. A. Rendell, T. S. Hoyne (remedies having a curative effect in skin diseases); P. Alvarez, Madrid; J. E. Winans, W. M. James, A. Fellger, C. W. Butler, C. Carleton Smith (comparative study of Lachesis and Lycopodium.)

Several of the above are engaged in the proving of Lac felinum.

### BUREAU OF CLINICAL MEDICINE.

Dr. J. A. Bigler, *Chairman;* Drs. Clement Pearson, clinical cases; Julius Schmitt, R. R. Gregg, clinical cases; A. McNeil, cases cured by Psoreinum; Ed. Bayard, nosodes; Benjamin Ehrman, Daniel W. Clauson, John Hall, F. Bruns (chronic diseases, how treated and crowned with success when we follow Hahnemann and the pioneers); E. W. Berridge.

Place of meeting: Washington, D. C.

NOTICE.

***All articles for publication, books for review, business communications, etc., must be sent to Editor, 2109 Chestnut Street, Philadelphia.***

